# CNN-Based Fault Detection of Scan Matching for Accurate SLAM in Dynamic Environments

**DOI:** 10.3390/s23062940

**Published:** 2023-03-08

**Authors:** Hyein Jeong, Heoncheol Lee

**Affiliations:** Department of IT Convergence Engineering, Kumoh National Institute of Technology, Gumi 39177, Republic of Korea

**Keywords:** deep learning, scan matching, 2D SLAM

## Abstract

This paper proposes a method for CNN-based fault detection of the scan-matching algorithm for accurate SLAM in dynamic environments. When there are dynamic objects in an environment, the environment that is detected by a LiDAR sensor changes. Thus, the scan matching of laser scans is likely to fail. Therefore, a more robust scan-matching algorithm to overcome the faults of scan matching is needed for 2D SLAM. The proposed method first receives raw scan data in an unknown environment and executes ICP (Iterative Closest Points) scan matching of laser scans from a 2D LiDAR. Then, the matched scans are converted into images, which are fed into a CNN model for its training to detect the faults of scan matching. Finally, the trained model detects the faults when new scan data are provided. The training and evaluation are performed in various dynamic environments, taking real-world scenarios into account. Experimental results showed that the proposed method accurately detects the faults of scan matching in every experimental environment.

## 1. Introduction

**Figure 1:**
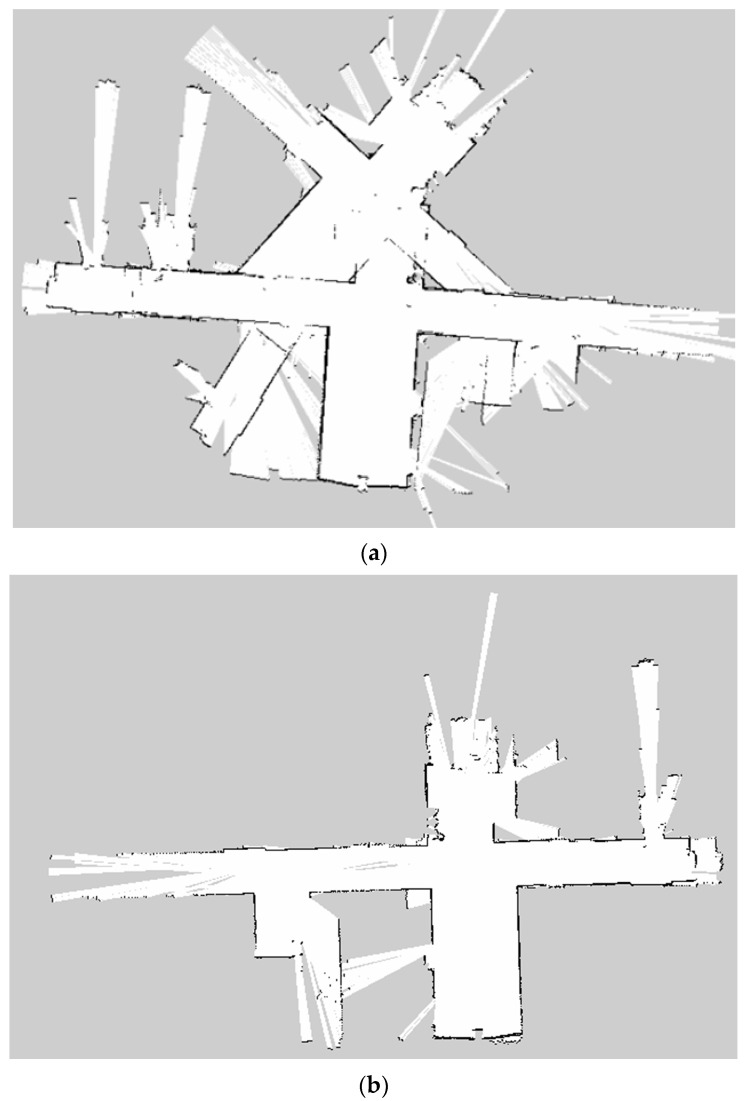
An example of the accuracy degeneration of SLAM in dynamic environments (**a**) SLAM in dynamic environments; (**b**) SLAM in static environments.

SLAM (Simultaneous Localization and Mapping) is a method which allows a mobile robot to build a map and estimate its location simultaneously in an unknown environment [1,2,3]. Despite recent studies in novel SLAM algorithms, SLAM in dynamic environments is still being studied for its more robust performance [4,5]. In 2D SLAM, when objects are dynamic or interrupt the light from a LiDAR (Light Detection and Ranging) in an environment, the detected environment by the sensor changes by seconds [6,7]; thus, the accuracy of SLAM degenerates, as shown in Figure 1.

SLAM problems in dynamic environments have been explored with various solutions. The authors of Ref. [8] proposed a filter-based dynamic object detection and removal method that requires both a camera and a laser scanner for visual SLAM and laser-based robot path planning. Ref. [9] suggested a distance filter to eliminate dynamic objects in laser scan data with multi-layer searching [10] for robot localization for 2D SLAM. Meanwhile, various methods utilizing deep learning for SLAM have been proposed. Ref. [11] is a visual SLAM with the LIFT network used to extract features of challenging environments. Ref. [12] suggested a moving object tracking algorithm that utilizes ML-RANSAC and CNN for SLAM in a dynamic environment. Ref. [13] suggested a SLAM system that uses local features obtained with CNN instead of traditional hand-made features. Regarding deep learning-based 2D SLAM, most existing methods use a method to convert range values of laser scans in Polar coordinates to panoramic grayscale images for the training of CNN models [14,15,16]. In this paper, for the training processes, we have used a new form of images which represent the laser range values as scan points in Cartesian coordinates with multiple colors. Table 1 compared the related works in the aspects of dynamic objects, the use of deep learning, and the type of sensors used.

As described previously, most of the existing SLAM solutions in dynamic environments or those which are deep learning-based require vision sensors to perform object detection for visual SLAM. An undesirable consequence of this is that the image data have a large data size and take much more time to process than laser scan data. In this regard, solutions for SLAM problems in dynamic environments with only a LiDAR, while not using vision sensors, are worth further exploration. Additionally, deep learning applications for 2D SLAM are still open to be thoroughly studied.

The main contributions of this paper are as follows.

**Figure 2:**
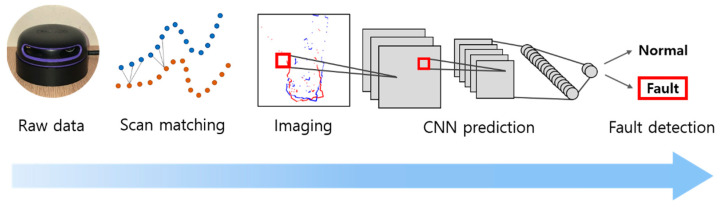
The concept overview of the proposed method. As soon as the raw scan data are received, the process of scan matching, the generation of images of matched scans, and fault detection are performed sequentially in each time step.

- An online process which includes raw scan data acquisition, scan matching, matched scan image generation, and the fault detection of the scan matching has been proposed and performed successfully, as shown in Figure 2.
- A method to form the training images which represent two consecutive laser scans, thereby taking advantage of the effective CNN-model training, has been proposed for the first time.
- The fault detection of scan matching has been conducted with high accuracy in various dynamic real environments.

The remainder of this paper is organized as follows: In Section 2, we describe the problem of deterioration of SLAM and scan matching in dynamic environments. Section 3 proposes the process of training image generation from raw scan data and a CNN model for the fault detection of the scan matching. Section 4 describes the experimental environments and reports the results. Finally, we conclude in Section 5.

## 2. Problem Description

### 2.1. SLAM in Dynamic Environments

Various 2D SLAM algorithms with a LiDAR have been developed. This work has utilized the GMapping algorithm, one of the widely used SLAM algorithms based on RBPF (Rao-Blackwellized Particle Filter), to build a 2D grid map and a robot trajectory [17,18]. During the filtering process, the estimated robot trajectory is defined as $p(x_{1:t}|z_{1:t},\;u_{1:t-1})$, where x is the robot position, z is the sensor observation, and u is control information from the robot odometer. Then, while using the estimated robot positions and observations from the sensor, the estimated map m is defined as: $p(m|x_{1:t},\;z_{1:t})$.

**Figure 3:**
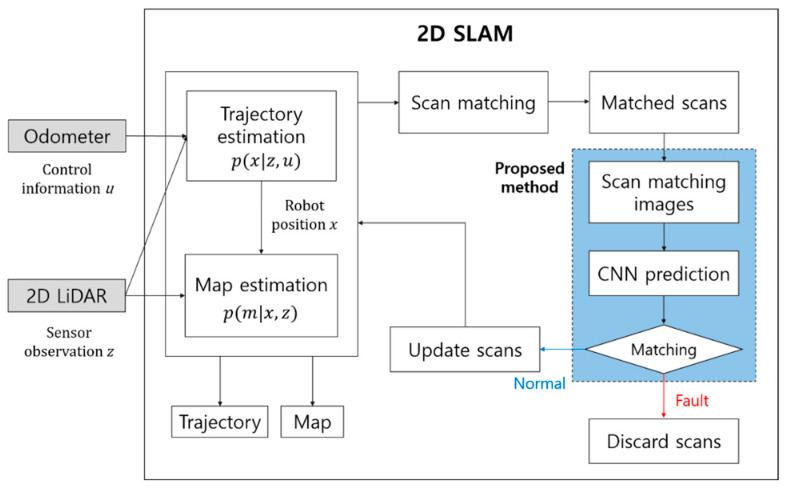
The whole structure of 2D SLAM with the proposed method.

However, in dynamic environments, the sensor data for the computations of the two posterior probabilities of the robot trajectory and the map are disrupted, resulting in bad SLAM performance. To improve this situation, our proposed method detects the faults of scan matching for the final decision to reflect the resulting scans from scan matching or otherwise on the computations as described in Figure 3, which represents the whole structure of 2D SLAM using the proposed method.

In 2D SLAM, a new laser scan is matched to a previous scan. To take the results of matching, the matched scans are converted into scan images. When the trained CNN model confirms that the scan matching is successful, the new laser scan is used for updates of the robot position and the map. If the scan matching is predicted to be faulty, the new laser scan is discarded, but only the odometer data at the time are used. Not using the unaligned scan points prevents an incorrect estimation of the map and the robot trajectory. 

### 2.2. ICP (Iterative Closest Points)

ICP is a scan-matching method that iteratively matches two consecutive laser scans. A transformation matrix that minimizes the distance between two scans is needed to align the current scan with the previous scan.

The closest points $p_{i}$ in the current scan to the points $q_{i}$ in the previous scan are searched. A cross-covariance matrix *K* is defined as:

(1) $$K=\overset{N}{\underset{i=1}{\sum }}p_{i}\cdot q_{i}{}^{T}=\left[\begin{array}{cc} cov\left(P_{x},\;Q_{x}\right) & cov\left(P_{x},\;Q_{y}\right)\\ cov\left(P_{y},\;Q_{x}\right) & cov\left(P_{y},\;Q_{y}\right) \end{array}\right]$$

Then, to find the rotation elements in the transformation matrix, K is decomposed as: $K=U\Sigma V^{T}$ by a singular value decomposition, where U is the left singular vector and V is the right singular vector. The rotation matrix R is defined as: $R=UV^{T}$. The transformation matrix is the combination of rotation and translation elements. Thus, the translation matrix t is defined as: $t=\mu_{Q}-R\cdot \mu_{P}$, where $\mu_{Q}\;$ and $\mu_{P}\;$ are the centers of mass of the previous scan and current scan. The rotation and translation matrix are iteratively computed until the scan matching error defined as: $err=∥\mu_{Q}-R\cdot \mu_{p}∥$, is smaller than a preset threshold. In this paper, the maximum number of iteration times and the error threshold were set as 20 and 0.01 (m), respectively. Disallowing too many iterations, the matching was terminated when the distance between two scans was less than 1cm. However, in this paper, most of the matchings were not finished before 20 iterations because the scan data were collected in environments which were dynamically set.

### 2.3. The Problem of Scan Matching in Dynamic Environments

**Figure 4:**
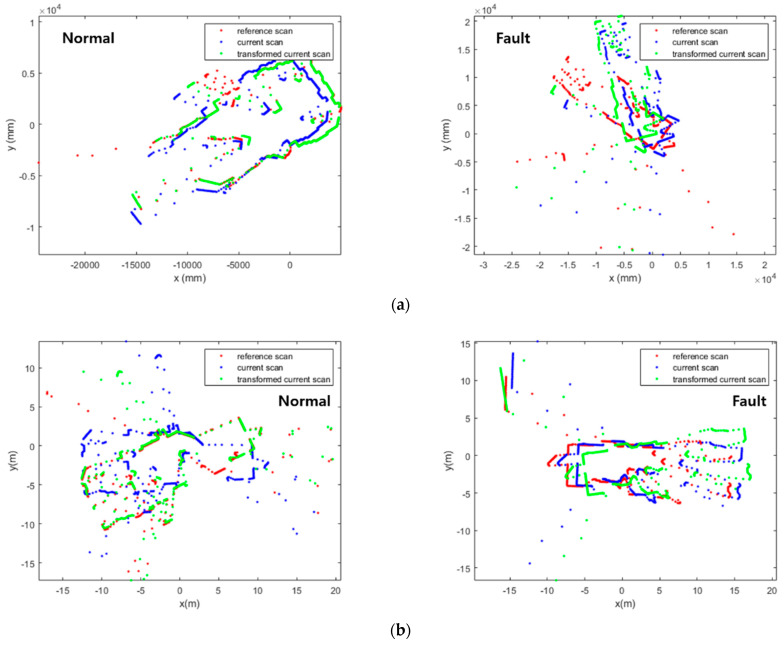

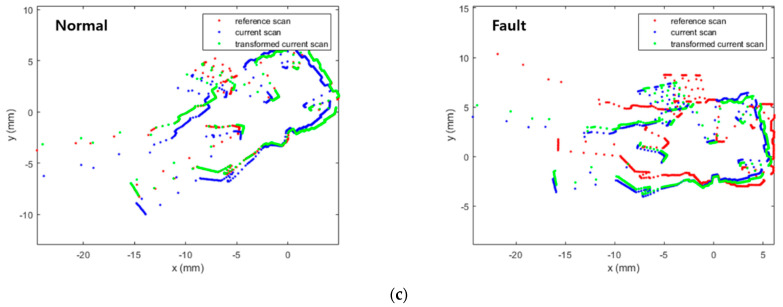
Normal and faulty scan matching with dynamic objects. (**a**) ICP (Iterative Closest Points); (**b**) PSM (Polar Scan Matching); (**c**) NDT (Normal Distributions Transform).

Scan matching is an important technique which allows SLAM to match the current scan to the previous scan of two consecutive laser scans by searching the overlapping parts [19]. ICP (Iterative Closest Points) [20,21,22,23,24], PSM (Polar Scan Matching) [25,26,27], and NDT (Normal Distributions Transform) [28,29,30] are representative scan matching methods. However, in dynamic environments, objects which move over time lessen the resemblance of two consecutive scans, making it challenging to perform accurate scan matching, as shown in Figure 4. In GMapping, scan matching is used to compute the maximum likelihood of the current robot position. The corrected robot position achieved via scan matching is defined as: $x_{t}=argmax\{p(z_{t}|x_{t},\;m_{t-1})\;p(x_{t}|u_{t},\;x_{t-1})\}$. However, scan matching may fail in dynamic environments, since the sensor data for the computations of the two posterior probabilities of the robot trajectory and the map are disrupted.

Consequently, the scan matching in GMapping, as well as the traditional scan matching methods, can be faulty due to dynamic objects and may cause errors in robot position estimation and map building. Even though various methods have been developed to resolve the problem of scan matching in dynamic environments, this remains an ongoing problem in SLAM communities. Therefore, this paper proposes a practical and reliable method to resolve the problem by detecting faulty scan matching based on deep learning.

## 3. Proposed Method

In environments with various dynamic scenarios, the raw scan data are acquired by a 2D LiDAR. The scans are then matched with ICP (Iterative Closest Points) scan matching. The matched scans are converted into scan images to be fed as training data for the CNN model for fault detection. When new scan data are acquired, the trained model detects faults in scan matching. The sequential tasks of acquisition of raw scan data, scan matching, conversion of matched scans into images, and fault detection are performed in a single process. 

### 3.1. Data Acquisition

**Figure 5:**
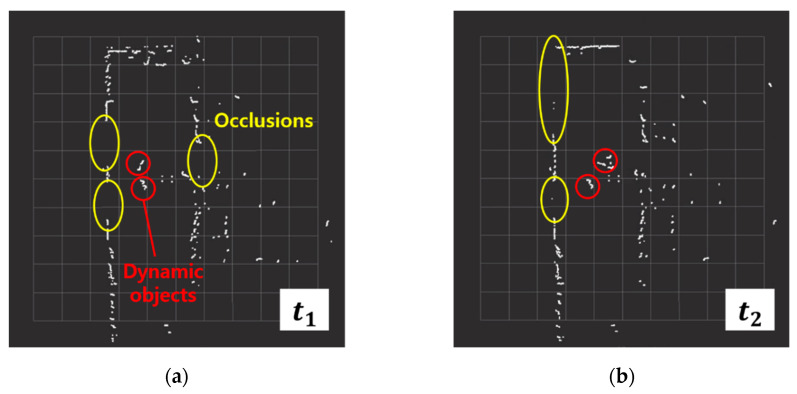

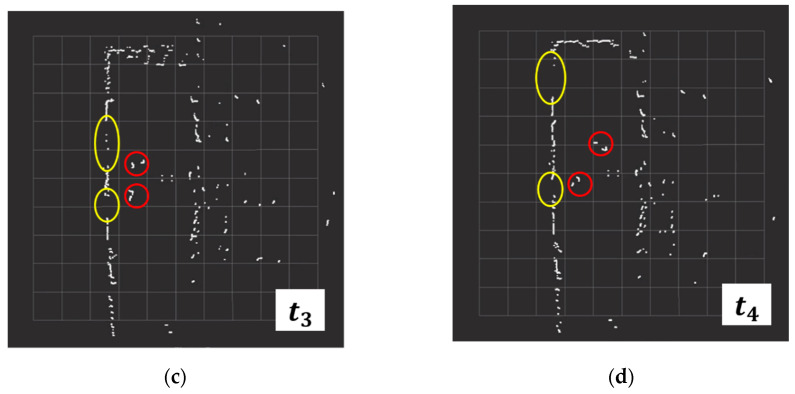
Dynamic objects and scan occlusions in raw scan data. The positions of objects and the occlusions of scans are changed over time. (**a**) t = *t*_1_; (**b**) t = *t*_2_; (**c**) t = *t*_3_; (**d**) t = *t*_4_.

To obtain a large amount of laser scan data for the training of the CNN model, raw scan data from a high-resolution LiDAR are received in ROS (Robot Operating System) sensor messages. The raw scan data include ranges values of dynamic objects and their scan occlusions, as shown in Figure 5.

### 3.2. Training Images

To conduct effective training of the CNN model, the scan points are converted into simple scan-matching images with clear features. To generate scan-matching images, two consecutive scans in the raw scan data are scan matched at each timestamp using ICP.

**Figure 6:**
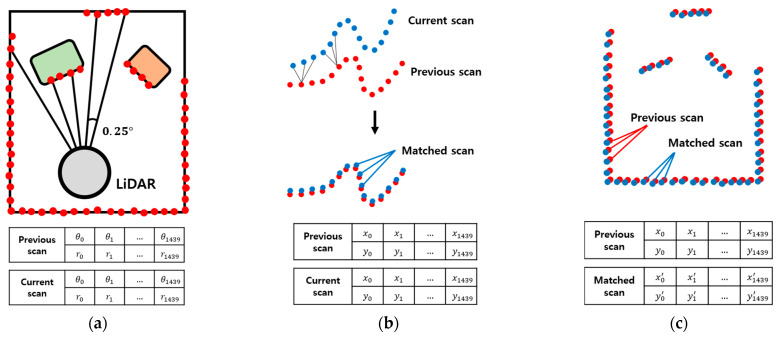
The generation of training images. (**a**) Raw scan range data in polar form; (**b**) ICP scan matching; (**c**) plotting of matched scans in Cartesian form.

**Figure 7:**
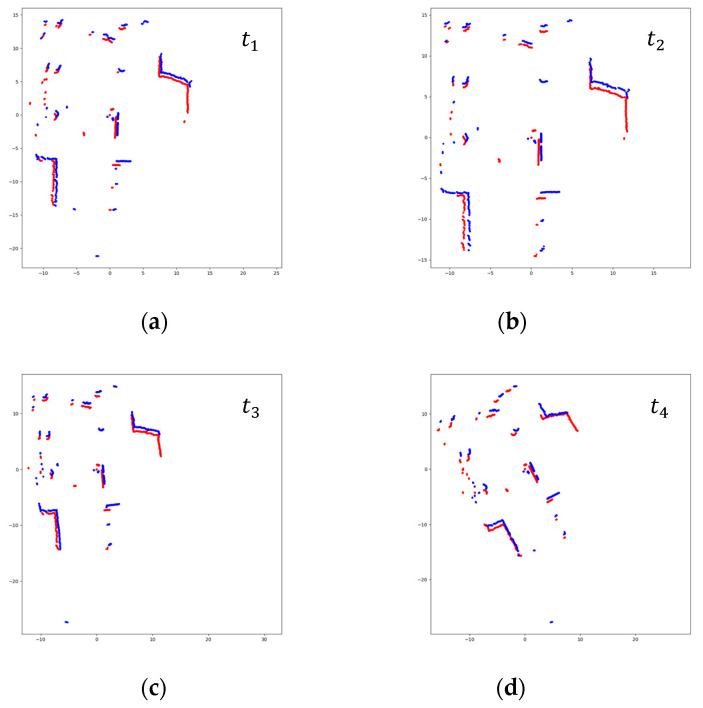
Conversion of scan pairs into images before preprocessing (Red: previous scan, blue: current scan). The areas where scans are plotted are not fixed. (**a**) t = *t*_1_; (**b**) t = *t*_2_; (**c**) t = *t*_3_; (**d**) t = *t*_4_.

The matched scans with Cartesian values are plotted as point graphs, as shown in Figure 6. To differentiate two scans in a pair, the previous scan is plotted in red, and the current scan in blue. Since the images contain only two scans in different colors on the white background, it is advantageous to extract features for the CNN model. In addition, to maximize the meaningful parts in images, the plotted areas of scan pairs are not fixed, but they change according to the ranges in which scans exist, as shown in Figure 7.

**Figure 8:**
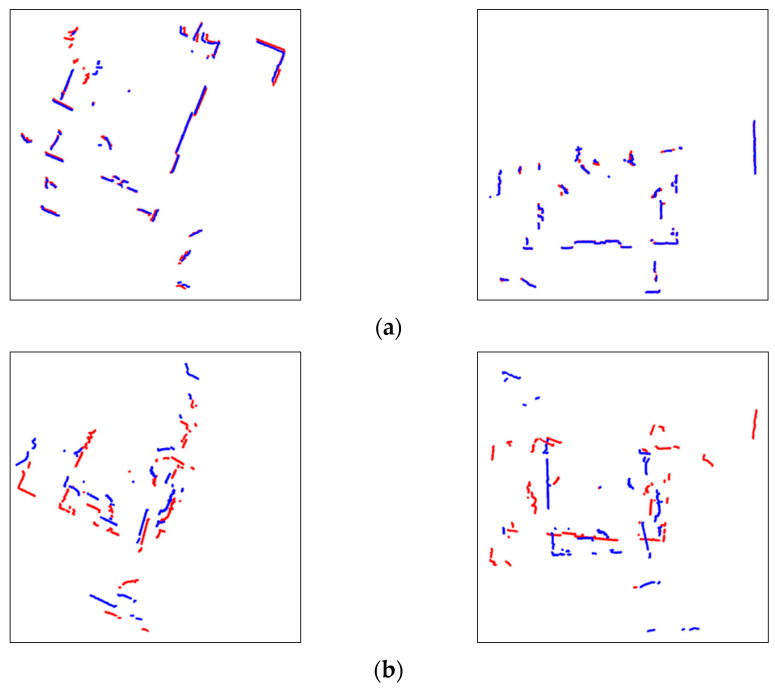
Classification of scan matching images after preprocessing (Red: previous scan, blue: current scan). (**a**) Normal; (**b**) fault.

For the preprocessing of plotted images, the unnecessary edge parts of scales are eliminated, and the sizes are reduced by about 1/16, to a final total of 256 × 256 pixels. The images are reduced in size for fast CNN model training while maintaining the features of scan matching. The processed images are automatically saved, then classified into binary class labels of normal and fault, as shown in Figure 8. The image generation and preprocessing were performed using matplotlib, a python visualization tool, which has a much shorter processing time than MATLAB. 

### 3.3. Fault Detection

**Figure 9:**
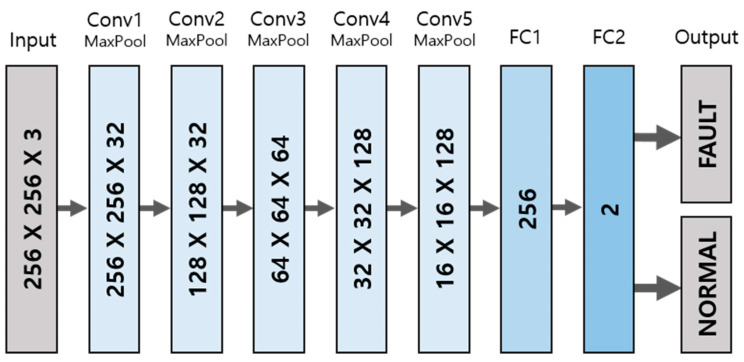
The architecture of CNN model for fault detection.

**Figure 10:**
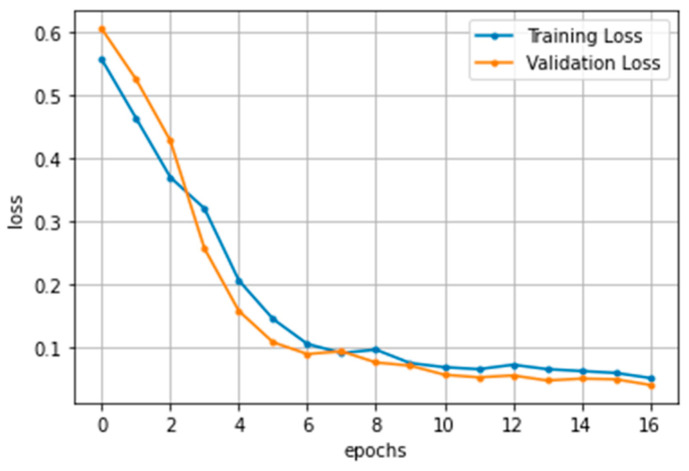
Training and validation loss of CNN model.

The CNN model used to perform the fault detection of scan matching has an architecture shown in Figure 9. For the model to distinguish two scans in different colors, the input images have 256 × 256 pixels with three dimensions of RGB. The raw pixel values of 0 to 255 are normalized in values between 0 and 1. For the feature extraction of images, five 2D convolutional layers are composed of the activation function ReLU (Rectified Linear Unit). Since the training images consist of primarily white backgrounds and limited areas of scan pairs, max-pooling layers are added for each convolutional layer to take meaningful values in feature maps. In addition, a quarter of neurons are dropped out after each max pooling to prevent over-fitting. Fully connected layers to flatten the extracted features follow the convolutional layers, resulting in the likelihood of class labels with the activation function Softmax; thereby, the scan matching can be found to be normal or faulty. For training and validation, binary_crossentropy loss for binary classification and Adam optimizer are utilized. The training epochs are set to 17, since the iteration number was sufficient to train images. Too much repetition of training may cause an over-fitted model. The batch size is set to 32 for the fast training; this value is not too large to affect the training accuracy. Both the training and validation loss desirably decrease during the training repetitions, as shown in Figure 10.

## 4. Experiments and Results

**Figure 11:**
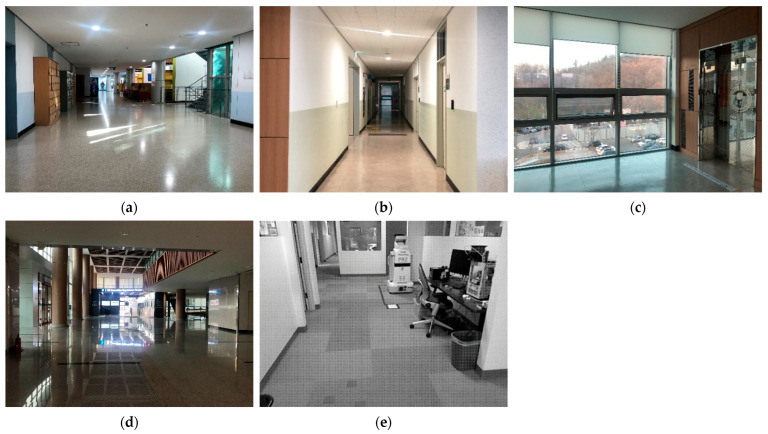
Experimental environments. (**a**) Environment 1; (**b**) Environment 2; (**c**) Environment 3; (**d**) Environment 4; and (**e**) Environment 5.

The environments for the experiments are set with multiple dynamic scenarios for an adaptive CNN model, as shown in Figure 11. To show the feature of each environment clearly, the people have been excluded from the figure. Environment 1 is a lobby with dynamic objects and clear landmarks. Landmarks with features are advantageous for scan matching, but the dynamic objects disturb the sensor data. Environment 2 is a narrow corridor with few features. This generally degrades the performance of scan matching. Environment 3 is an indoor environment with mirrors (lifts) and windows; this makes the range values from the sensor unreliable. Environment 4 is a wide hall; its structure partially exceeds the detection distance of LiDAR. In addition to these challenging conditions, Environments 1, 2, 3, and 4 were stuffed with dozens of people. Lastly, the Willow Garage dataset [31] was used as Environment 5. The environment is an indoor office with a few moving objects. For precise validation of the performance, the data collected in environment 5 were not used for training, but only to test the trained model.

**Figure 12:**
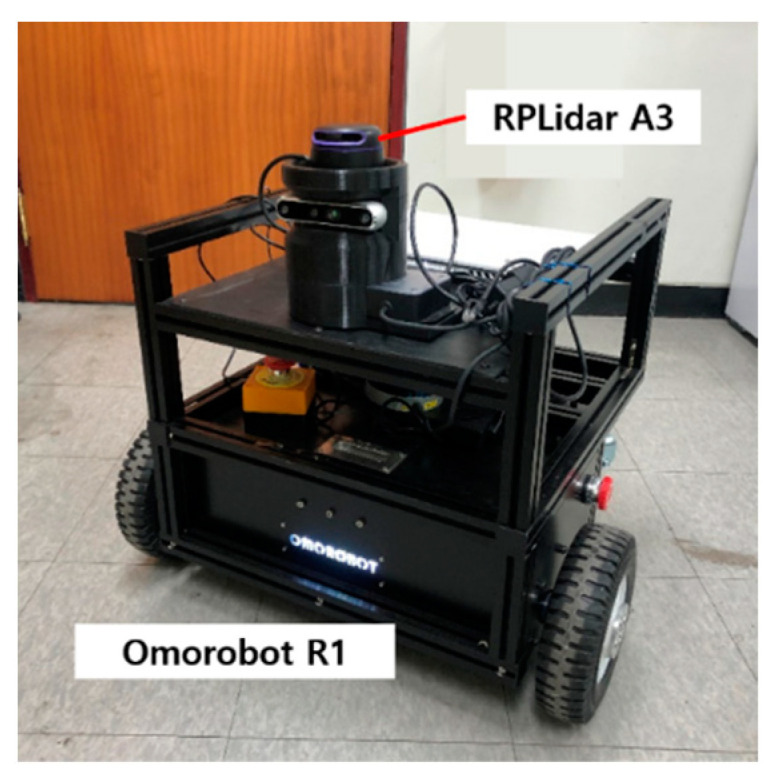
Robot and sensor used for experiments.

The mobile robot used for experiments is Omorobot R1, which has a maximum speed of 1.2 m/s and an odometer for the control information, as shown in Figure 12. For the detection of environments, RPLidar A3 is used with a maximum detection distance of 25 m, taking 1440 points for each scan with 360° of FoV (Field of View). With the sensor with high angular resolution (0.25°), acquiring large amounts of data is possible. The Willow Garage dataset has exceptive data, of which laser scans have 1040 points with 270° of FoV. 

### 4.1. Scan Matching

**Figure 13:**
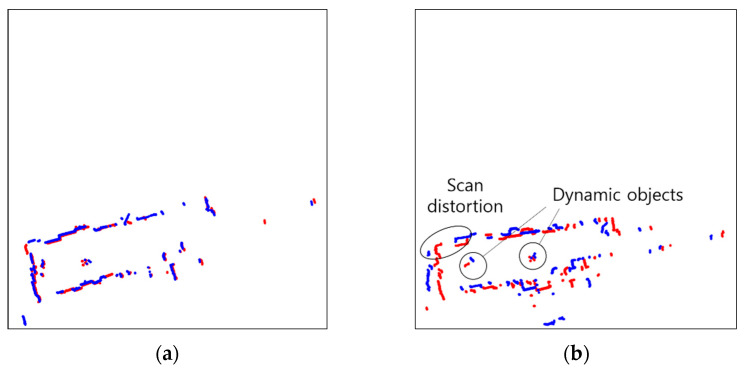
Scan matching images in environment 1 (Red: Previous scan, blue: current scan). (**a**) Normal matching images; (**b**) faulty matching images.

**Figure 14:**
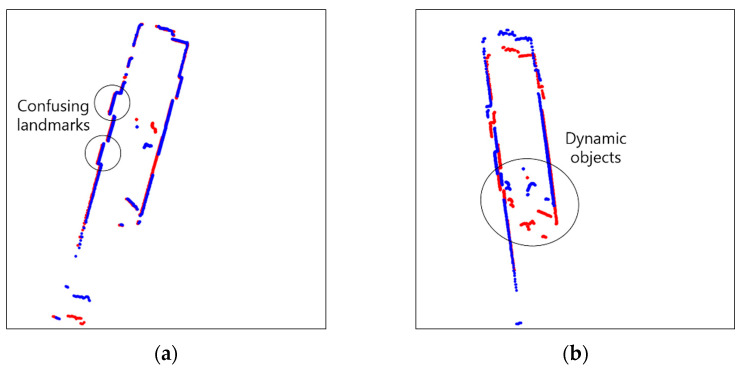
Scan matching images in environment 2 (Red: Previous scan, blue: current scan). (**a**) Normal matching images; (**b**) faulty matching images.

**Figure 15:**
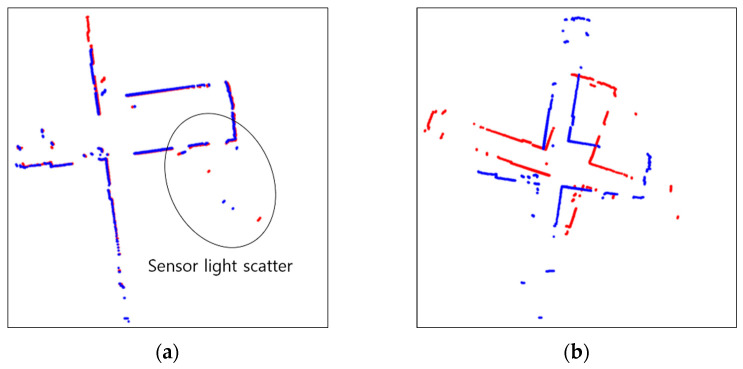
Scan matching images in environment 3 (Red: Previous scan, blue: current scan). (**a**) Normal matching images; (**b**) faulty matching images.

**Figure 16:**
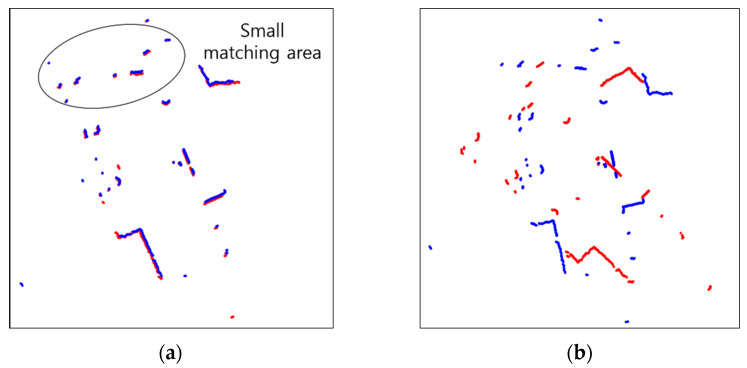
Scan matching images in environment 4 (Red: Previous scan, blue: current scan). (**a**) Normal matching images; (**b**) faulty matching images.

**Figure 17:**
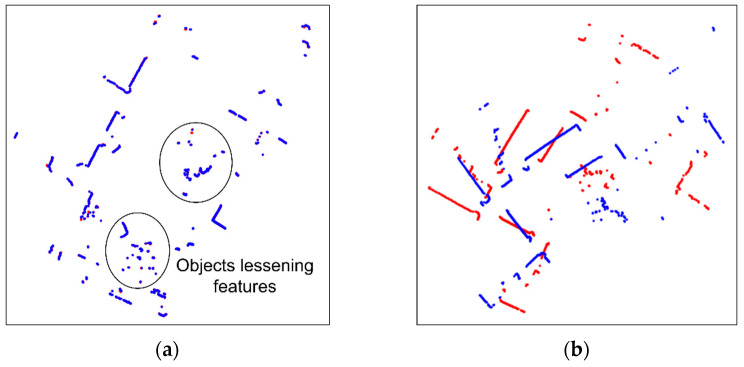
Scan matching images in environment 5 (Red: Previous scan, blue: current scan). (**a**) Normal matching images; (**b**) faulty matching images.

The results of scan matching in Environments 1, 2, 3, 4, and 5 are shown in Figure 13, Figure 14, Figure 15, Figure 16 and Figure 17. In Environment 1, despite landmarks that provide good features for scan matching, dynamic objects disturb the detection of LiDAR. As a result, scan matching occasionally fails. Scan matching in Environment 2 is more challenging than in Environment 1, which has few landmarks except the walls of the corridor. This is especially true when the mobile robot moves in a straight line without rotation, as the consecutive scans from the motion still have large overlapping areas of walls before scan matching. That is, the structural features which are straight and continuous can stop the iterations of scan matching, even though the scans are not yet adequately matched. When objects are added to this situation, the performance of scan matching deteriorates further. Environment 3 shows the faults of scan matching caused by both dynamic objects and the discouraging factors of sensor detection. The mirrors (lifts) reflect the sensor light; thus, the scan points representing the structure are scattered. Likewise, windows allow light to pass through them; therefore, the sensor detection becomes unreliable and the environment map highly deteriorates. In Environment 4, the scan points on the parts which are out of the range of the sensor are received as infinite values, and the values are discarded for scan matching. This results in small matching areas, and the areas are further reduced by dynamic objects, which hide the landmarks in the environment from LiDAR detection. Environment 5, the public Willow Garage dataset, has data with a smaller detection angle than the others, and many objects hide a large part of its original structure.

### 4.2. Fault Detection

For training and testing, among 20,830 images in total, 13,254 normal images and 7576 faulty images are used at a ratio of three (training) to one (testing). For the validation of the prediction, 2969 normal images and 1283 faulty images of 4252 images are used. Data from all environments described in 4.1 are used for the training and testing, and the validations of label prediction are conducted using the total data of all environments and the respective data of each environment. Table 2 shows the confusion matrix for prediction results of fault detection in various environments.

Environment 1 shows the highest accuracy in detecting faults of 99.6%. This is because the images in this environment more clearly show whether the two consecutive scans are aligned or not, compared to other environments. For the fault detection of the trained model, the mismatch of scans results in more red points of the previous scan in an image. That is, since the mismatched red points in Environment 1 are clearly shown in faulty scan-matching images, the fault detection is successful. Comparatively, in Environment 2, the fault detection has 99.3% accuracy, which is the lowest accuracy. Because the faults of scan matching in the environment are mostly linearly unaligned, the current scan in blue covers the most of the previous scan in red, but the two scans are not yet matched. These faults less clearly show the unconformity of the scans on images than faults in other environments, making the fault detection of the CNN model confusing. However, these errors are reduced by feeding a large number of linearly faulty images; thereby, the trained model is still robust in its predictions. Environment 3 shows good results, except for four incorrectly labeled predictions among 998 images, resulting in 99.5% accuracy. Due to the windows and mirrors, as well as dynamic objects, the red previous scan points partially decrease. The scan matching errors in this situation are wrongly predicted, but they are handled by training data from different trajectories and environments. Likewise, in Environment 4, the scan points of two laser scans are reduced by its spacious structure and objects. The images of this matching error contain fewer features, which demonstrates the quality of scan matching. Still, the trained model predicts it with an accuracy of 99.5%. Only a few slightly unaligned scan images are incorrectly predicted. Environment 5 is used only to test the trained model, and is never used for the fault detection training. Even though the images reduce features as a result of the objects, 98.9% accuracy is achieved due to the large dataset taken from Environments 1–4.

**Figure 18:**
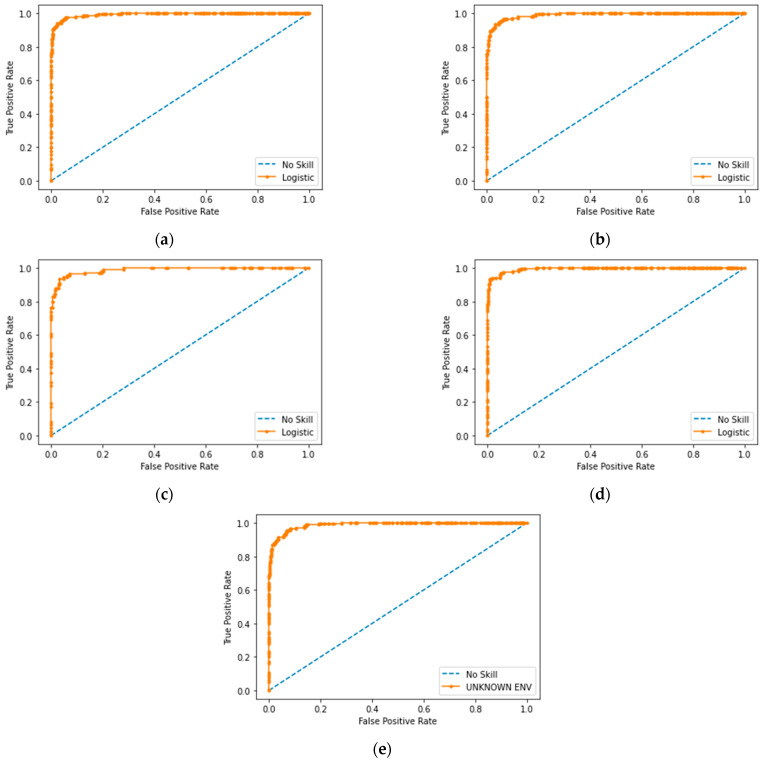
ROC curve of prediction in (**a**) Environment 1; (**b**) Environment 2; (**c**) Environment 3; (**d**) Environment 4 and (**e**) Environment 5.

**Figure 19:**
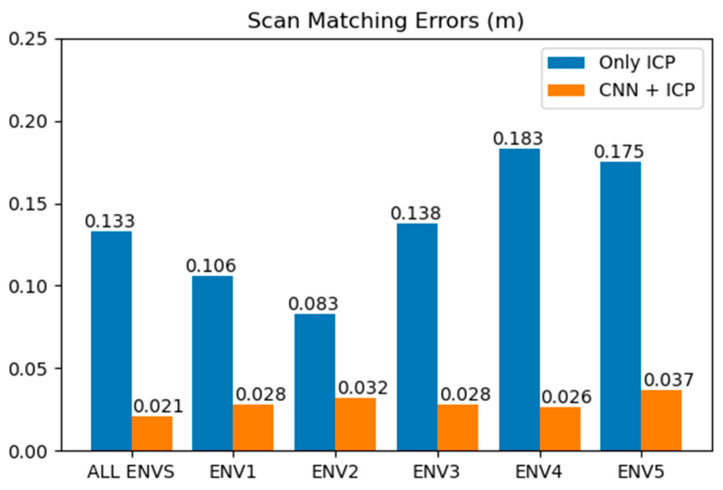
Comparison of methods in terms of scan matching error. The error is Euclidian distance of matched points, which represents the misalignment of scans.

The results are also presented as ROC (receiver operating characteristic) curves in Figure 18. As shown in the graphs, the trained CNN model accurately detects the faults of scan matching, regardless of the environment. The proposed method is adaptive for various environments with their own features. In addition, we compare the proposed method to the original ICP in terms of scan matching error, as shown in Figure 19. The error is defined as the Euclidian distance between the centers of mass of two matched scans, as described earlier in Section 2.2. The smaller the error, the more accurate the scan matching. The inaccuracy in matchings decreases by 75% using our CNN method. 

## 5. Conclusions

We have proposed an applicable CNN model for the fault detection of scan matching to complement SLAM in dynamic environments. For fault detection, as soon as the raw scan data are received, scan matching, the conversion of matched scans into images, and the fault detection by the trained CNN model are performed sequentially at each time step. The scan data for the training of the model are acquired under various dynamic scenarios to compare the model’s performances in real-world environments. As the training data for a CNN model, the form of images that represent consecutive scan data in Cartesian coordinates are used for the first time and demonstrate efficiency in the model training. The validation of the trained model shows accuracy of over 99% in the detection of faults of scan matching in every experimental environment.

For future work, the method can be extended to various SLAM frameworks in real time. Depending on the methods of scan matching, mapping, and robot pose estimation of different SLAM algorithms, the steps of scan matching and the application of fault detection in the proposed method can be adapted to enhance existing SLAM and scan registration systems.

## Figures and Tables

**Table 1:** The comparison of related works.

Reference	Dynamic Objects	DL-Based	Sensors
Proposed	O	O	Only LiDAR
[8]	O	X	Vision, LiDAR
[9]	O	X	Only LiDAR
[11,13]	X	O	Vision
[12]	O	O	Vision, LiDAR
[14,15]	X	O	Only LiDAR
[16]	X	O	LiDAR, IMU

**Table 2:** Predictions of the proposed method.

	TP	FN	FP	TN	Recall	Precision	Accuracy
All environments	2946	23	6	1277	99.2%	99.7%	99.3%
Environment 1	978	3	2	274	99.6%	99.7%	99.6%
Environment 2	745	4	3	352	99.4%	99.4%	99.3%
Environment 3	700	3	1	294	99.5%	99.8%	99.5%
Environment 4	534	2	2	355	99.6%	99.6%	99.5%
Environment 5	702	7	4	360	98.0%	99.4%	98.9%

## Data Availability

Not applicable.
